# A Multi-Age-Group Interrupted Time-Series Study for Evaluating the Effectiveness of National Expanded Program on Immunization on Mumps

**DOI:** 10.3390/vaccines10101587

**Published:** 2022-09-21

**Authors:** Chen Shi, Wen-Hui Liu, Lin Yang, Ze-Lin Yan, Li Li, Zhou-Bin Zhang, Chun-Quan Ou

**Affiliations:** 1 State Key Laboratory of Organ Failure Research, Department of Biostatistics, Guangdong Provincial Key Laboratory of Tropical Disease Research, School of Public Health, Southern Medical University, Guangzhou 510515, China; 2 Guangzhou Center for Disease Control and Prevention, Guangzhou 510440, China; 3 School of Nursing, Hong Kong Polytechnic University, Hong Kong 999077, China

**Keywords:** mumps, mixed-effects quasi-Poisson regression model, the national Expanded Program on Immunization, interrupted time series, China

## Abstract

The national Expanded Program on Immunization (EPI) in China has covered vaccines for measles, mumps, and rubella, among children aged 18–24 months since September 2008. However, no previous studies have quantified the effectiveness of the EPI on mumps incidence. There are methodological challenges in assessing the effect of an intervention that targets a subpopulation but finally influences the whole population. In this study, monthly data on mumps incidence were collected in Guangzhou, China, during 2005–2019. We proposed a multi-age-group interrupted time-series design, setting the starting time of exerting effect separately for 14 different age groups. A mixed-effects quasi-Poisson regression was applied to analyze the effectiveness of the EPI on mumps incidence, after controlling for long-term and seasonal trends, and meteorological factors. The model also accounted for the first-order autocorrelation within each age group. Between-age-group correlations were expressed using the contact matrix of age groups. We found that 70,682 mumps cases were reported during 2005–2019, with an annual incidence rate of 37.91 cases per 100,000 population. The effect of EPI strengthened over time, resulting in a decrease in the incidence of mumps by 16.6% (EPI-associated excess risk% = −16.6%, 95% CI: −27.0% to −4.7%) in September 2009 to 40.1% (EPI-associated excess risk% = −40.1%, 95% CI: −46.1% to −33.3%) in September 2019. A reverse U-shape pattern was found in age-specific effect estimates, with the largest reduction of 129 cases per 100,000 population (95% CI: 14 to 1173) in those aged 4–5 years. The EPI is effective in reducing the mumps incidence in Guangzhou. The proposed modeling strategy can be applied for simultaneous assessment of the effectiveness of public health interventions across different age groups, with adequate adjustment for within- and between-group correlations.

## 1. Introduction

Mumps is an infectious disease caused by the mumps virus that causes puffy cheeks and swollen jaw [1]. Serious consequences, such as orchitis, oophoritis, pancreatitis, hearing loss, meningitis, and encephalitis, can develop after mumps virus infection [2]. Globally, mumps incidence ranged from 100 to 1000 cases per 100,000 persons of the entire population, in the absence of vaccination [3]. In China, a total of 909,087 mumps cases were reported accumulatively during 2008–2010, with an annual average incidence of 22.8 cases per 100,000. Up to 81.8% of the cases occurred in children aged 3–14 years and 97.0% of the outbreaks occurred in child care settings and schools, especially in primary schools [4]. The best approach to prevent mumps is to be vaccinated. Since the 1980s, more than 60% of WHO member states have included mumps vaccination in their national immunization programs, reducing the frequency of mumps and severe complications [5,6]. In mainland China, before 2008, mumps vaccines were not included in the national program on immunization, implying that parents had to pay out-of-pocket for a mumps vaccine. As a result, the mumps vaccination rate was low because mumps vaccination was self-supported and voluntary at that time [7]. Since 2008, the Expanded Program on Immunization (EPI) has provided free one-dose combined vaccine against measles, mumps, and rubella (MMR) for children aged 18–24 months [8,9] (EPI-MMR represents this intervention in the remaining of the paper).

Evidence on the effectiveness of vaccination programs can support and guide vaccination promotion, particularly in countries where the mumps vaccine for children program has not been implemented. Although a few studies have demonstrated the effectiveness of vaccination campaigns in other countries, such as the Netherlands [10,11], data from more regions are needed to support the advancement of the program due to differences in vaccination strategies, as well as populations. In addition, there are still some gaps in the existing studies. Some prior epidemiological studies described the average annual incidence rate between pre-EPI-MMR and post-EPI-MMR or between the durations specified [12,13,14]. However, these studies could not draw a conclusion on the causal effect of the EPI-MMR on the averted number of mumps cases, since there were also many other time-varying variables including environmental, socio-economic, and healthcare-related factors, that may affect mumps incidence [15,16,17], especially if the procedures were carried out over an extended period of time. Some researchers have investigated the effectiveness of mumps-containing vaccinations in an indirect manner by case-control studies or cohort studies [4,18]. However, these studies involved subjects based on specific inclusion and exclusion criteria and therefore the preventable number of mumps cases cannot be estimated for the whole population.

The interrupted time-series (ITS) approach has been widely used to analyze the effects of public health programs [19]. However, most studies did not consider data autocorrelation across time and age cohorts [20,21]. However, the EPI-MMR in China is an intervention targeting children aged 18–24 months, not for the entire population; therefore, different birth cohorts would have differential benefits from the intervention. The overall effect depends on the age structure of the population. To evaluate the intervention effect on a specific subpopulation, a few studies performed subgroup analyses, or only included the intervened group in the ITS analysis [22], ignoring the intergroup correlation that particularly plays an important role in the incidence of infectious diseases. There are methodological challenges in evaluating intervention effect for multiple age groups after dealing with the issue of within-age-group temporal autocorrelation, and between-age-group correlations.

The purpose of this population-based study is to develop a multi-age-group ITS approach to evaluate the intervention effect of the EPI-MMR in Guangzhou, China. The findings would not only assist in the continuation or revision of the current mumps vaccination strategy, but also serve as an example for the future evaluation of policies.

## 2. Materials and Methods

### 2.1. Data Collection

We extracted the individual data of mumps cases occurring in Guangzhou between 1 January 2005 and 31 December 2019 from the National Infectious Disease Monitoring Information System, as compiled by the Guangzhou Center for Disease Control and Prevention. The information on each case included date of birth, age, date of diagnosis, and sex. The study included both clinically confirmed and laboratory-confirmed cases [23]. Individual data were aggregated into the monthly number of cases for each age group. 

The surface meteorological information was collected from China Meteorological Administration (http://data.cma.cn/ accessed on 30 April 2022). We collected the overall and age group-specific resident population by the end of 2000, 2010, 2015, and 2020, from Guangzhou Statistics Bureau (http://tjj.gz.gov.cn/ accessed on 30 April 2022). The daily resident population was obtained by linear interpolation. Finally, the month-by-month data were obtained by monthly averaging.

### 2.2. Study Design and Setting

We conducted an ecological study to evaluate the effects of the EPI-MMR on the entire population in Guangzhou, where high-quality and sustainable data of mumps cases was available before and after the EPI-MMR. Given that the EPI-MMR was implemented for children aged 18–24 months since September 2008, different birth cohorts were subjected to direct policy interventions at different time intervals. We divided the entire population into 14 age groups, including 0–1.5 years (unvaccinated), 1.5–2 years, and 11 one-year groups from 2 to 13 year (likely to be vaccinated), and 13+ years (unvaccinated during the study period). The starting time of the exerting effect of the EPI-MMR are specified separately for each age group. For 0–1.5 years, the EPI-MMR did not have a direct impact on this group during the study period of 2005–2019, therefore, the whole study period was pre-intervention (Figure S1). For 1.5–2 years, there was a pre-intervention period before September 2008. We did not expect all children aged 1.5–2 years to receive MMR vaccinations at once after the policy was implemented, hence the post-intervention period was defined as March 2009 to December 2019. That is, a six-month period (September 2008 to February 2009) was designated as a phase-in period and was left out of the analyses. The pre- and post-intervention period for other age groups were provided in Figure S1.

### 2.3. Statistical Analysis

A mixed-effects quasi-Poisson regression model, which accounted for the within- and between-age-group correlations, was used to assess the effects of the EPI-MMR on mumps incidence in Guangzhou, China.

$$\begin{array}{ll} \log\left(u_{it}\right) & =a_{i}+offset\left(\log\left(pop_{it}\right)\right)+\beta_{1}t+\beta_{2}X_{it}+\beta_{3}t\times X_{it}+\beta_{4}\overset{n}{\underset{j=1}{\sum }}W_{ij}y_{jt}\\ & +\overset{k}{\underset{\theta =1}{\sum }}\left[\beta_{1\theta }\sin\left(\frac{2\theta \pi mon_{m}}{T}\right)+\beta_{2\theta }\cos\left(\frac{2\theta \pi mon_{m}}{T}\right)\right]+ns\left(tem_{it},df=3\right)+ns\left(rh_{it},df=3\right)\\ & +ns\left(wind_{it},df=3\right)+ns\left(ssd_{it},df=3\right) \end{array}$$

where $u_{it}$ is the mean of the number of mumps cases in age group i in month $t\left(1,2,\ldots ,t_{s}-1,\ldots ,t_{e}+1,\ldots ,180\right)$, with a variance of $\varphi u_{it}$ conditional on $a_{i}$, the random intercept element that accounts for age-group heterogeneity. And $t_{s}$ is the start of phase-in period and $t_{e}$ is the end of phase-in period. And φ is the over-dispersion parameter. The logarithm of population, with a fixed regression coefficient of 1, was utilized as an offset. $X_{it}$ is a categorical variable with 0 and 1 indicating pre- and post-intervention period, respectively. The interaction term of $X_{it}$ and t expressed the time-varying effects of the EPI-MMR. To fit the seasonality of mumps incidence, the paired sine and cosine functions were applied; T is 12 and k was chosen by spectral analysis (k = 2) (Figure S2); $mon_{m}$ denotes the mth month of the year. $ns\left(tem_{it}\right)$, $ns\left(rh_{it}\right)$, $ns\left(wind_{it}\right)$, $ns\left(ssd_{it}\right)$ are natural cubic splines of monthly average temperature, average relative humidity, average wind speed and average duration of sunshine with three degrees of freedom (dfs).

**Figure A1:**
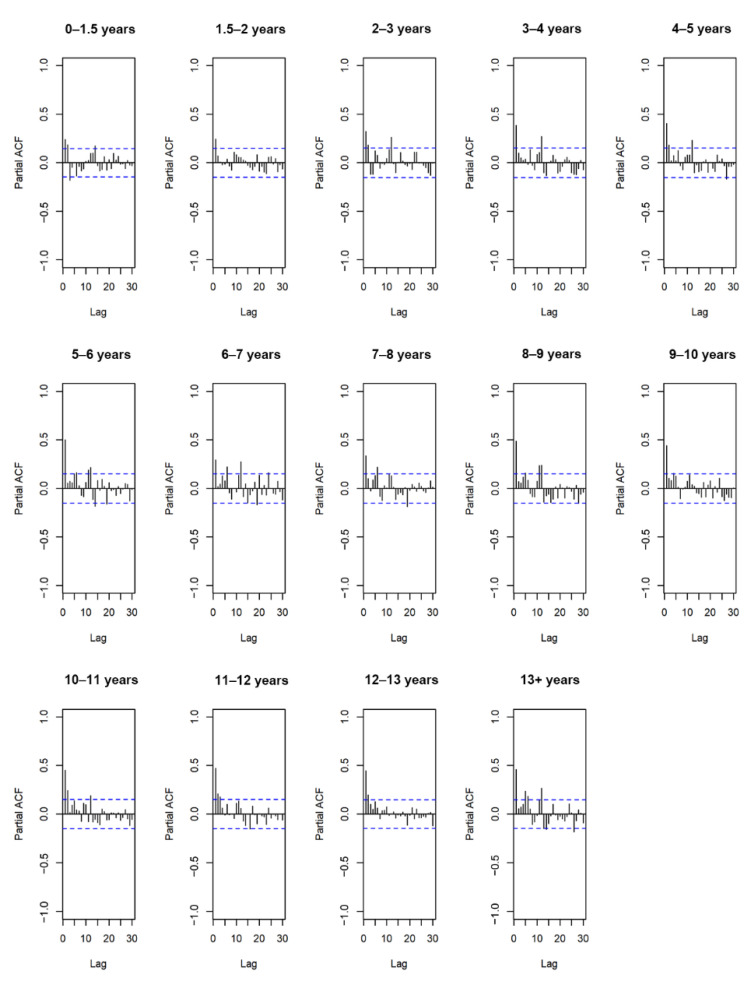
The partial autocorrelation functions of residuals from preliminary analyses for 14 age groups.

**Figure A2:**
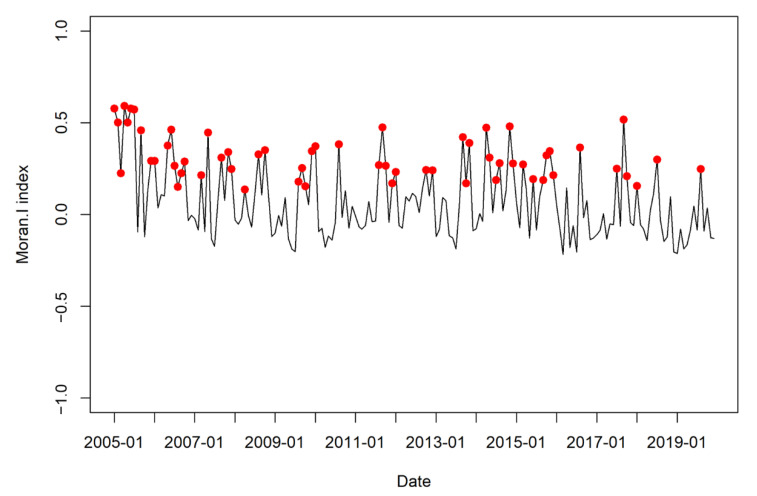
The Moran’s I index for 14 age groups from January 2005 to December 2019. Red points indicate there is significant between-groups correlation of residuals.

In our preliminary analyses (Appendix A), we found that the ordinary quasi-Poisson regression model demonstrated obvious temporal autocorrelation of residuals at lag1 in most age groups (Figure A1) and significant correlation between age groups (Figure A2). Therefore, in the final mixed-effects quasi-Poisson regression model, a conditional covariance matrix for autocorrelation was defined to account for the temporal autocorrelation within an age group. $\sum_{j=1}^{n}W_{ij}y_{jt}$ was used to express the between-age-group correlations. $W_{ij}$ denotes contact matrix between age group i and age group j. If i=j, the diagonal elements were set to zero. $y_{jt}$ is the number of mumps cases in age group j in month t. Here, the social contact data of Shanghai in 2019 (Table S1) [24] were adopted, since Guangzhou and Shanghai have similar socio-economic conditions. We checked the assumption of independence within each age group (temporally) and between age groups using PACF and the Moran’s I statistic for the final model. We did not find substantial between-group correlations (Figure S3) and the within-group autocorrelation issue was greatly improved (Figure S4).

We estimated excess risk (ER) and excess morbidity rate (EMR) to evaluate the effect of the EPI-MMR. The ER of mumps incidence at month t was expressed as ${\hat{ER}}_{t}=\left(\exp\left({\hat{\beta }}_{2}+{\hat{\beta }}_{3}\times t\right)-1\right)\times 100\%$. The EMR was estimated as: $\left(\sum_{t=t_{0}}^{t=t_{1}}\left({\hat{Y}}_{it}|(X_{it}=1)-{\hat{Y}}_{it}|(X_{it}=0)\right)/pop_{i}\right)\times 100,000/N_{i}$, where ${\hat{Y}}_{it}|(X_{it}=1)$ is the predicted number of mumps in post-intervention month t under the factual scenario ($X_{it}=1$); ${\hat{Y}}_{it}|(X_{it}=0)$ is the predicted number of mumps under the counterfactual scenario that the intervention was not carried out (i.e., $X_{it}=0$), and $N_{i}$ is the number of years after the intervention under study for age group i. Furthermore, 95% empirical confidence intervals (95% eCIs) were computed using the Monte Carlo simulation for the regression coefficients, assuming the coefficients followed a multivariate normal distribution.

To test the robustness of the main findings, we performed sensitivity analyses. First, we re-fitted the model by regarding the phase-in periods defined in the main analyses as one of a period of pre-intervention (CApre) or post-intervention (CApost), respectively. Second, we investigated the potentially nonlinear intervention effect over time by replacing $t\times X_{it}$ with $ns\left(t,df=3\right)\times X_{it}$. ns(t,df=3) denotes a natural cubic spline function of time t with three dfs. We used R statistical software (version 4.1.1) to perform all analyses.

## 3. Results

From January 2005 to December 2019, a total of 70,682 mumps cases were recorded in Guangzhou. The annual incidence rate was 37.91 cases per 100,000 people, on average. Most of the cases (63.4%) were males and children aged 5–9 years accounted for 40.5% of all cases. Mumps incidence declined from 2005 to a nadir point in 2007, peaked in 2011, and subsequently decreased. The number of recorded mumps cases declined by 77.7% from 10,500 persons in 2011 to 2344 persons in 2019. (Table 1)

**Figure 1:**
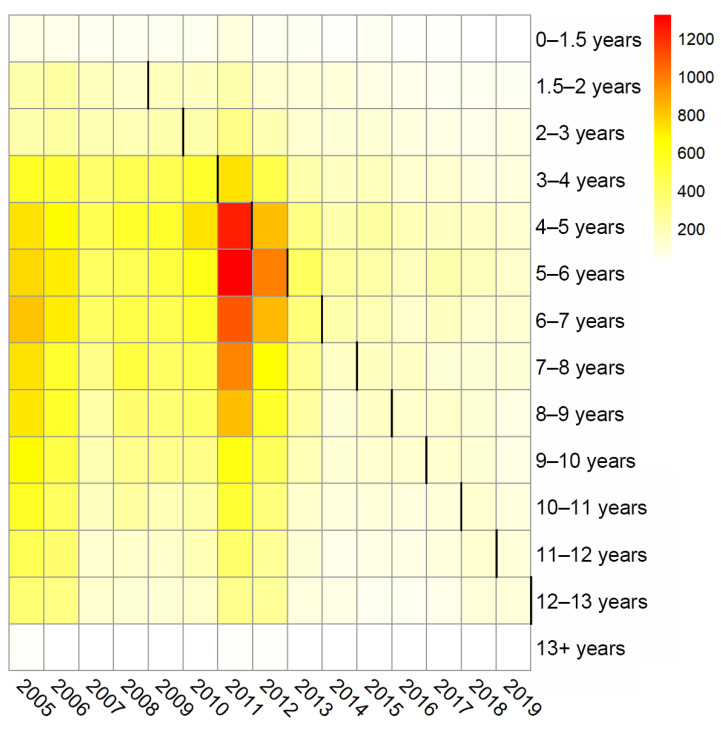
Heat map of the annual mumps incidence (per 100,000 population) for 14 age groups during 2005–2019. The black line indicates the year in which the intervention began for the age group.

The average yearly incidence rates of mumps during 2005–2019 for all of the 14 age groups are presented in Figure 1 and Table S2. The heat map indicated that the incidence rate in all age groups decreased to some extent after the intervention. Of these, 2012 is the most noteworthy; before 2012, there were substantial intergroup variations of mumps incidence rate, with the highest incidence rate in children aged 4–8 years. After 2012, the intergroup differences of mumps incidence rate were steadily narrowed, with the number of age groups with an annual incidence rate of less than 100/100,000 climbing from two in 2005 to nine in 2019 (Figure 1 and Table S2).

**Figure 2:**
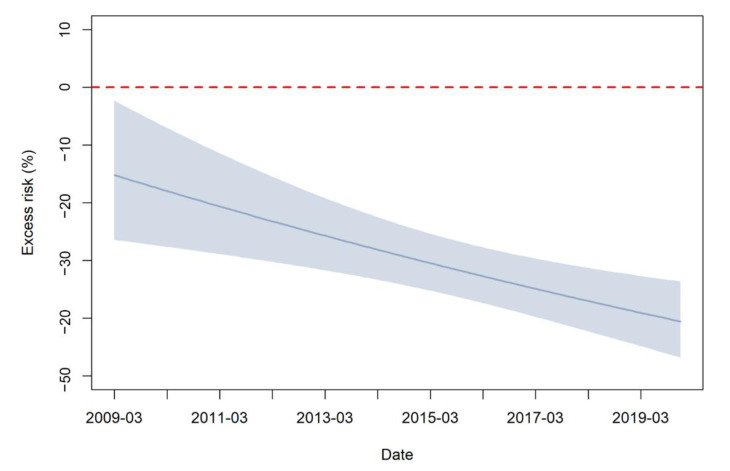
Excess risks of mumps incidence attributable to the EPI-MMR intervention in Guangzhou, China. The shadow represents the 95% confidence intervals of excess risks.

Figure 2 displays the estimates of ERs of mumps incidence associated with the EPI-MMR intervention from 2009–2019. The introduction of the EPI-MMR resulted in a decrease in the incidence of mumps from the beginning to 2019 after adjustment for long-term trend, seasonality, and meteorological measures, with a decrease of 40.1% (95% CI: 33.3% to 46.1%) (Table S3). The magnitude of intervention effect changed over time. In the first year after the intervention, the intervention effect was relatively weak (ER = −15.2%, 95% CI: −26.4% to −2.3%) and gradually strengthened.

**Figure 3:**
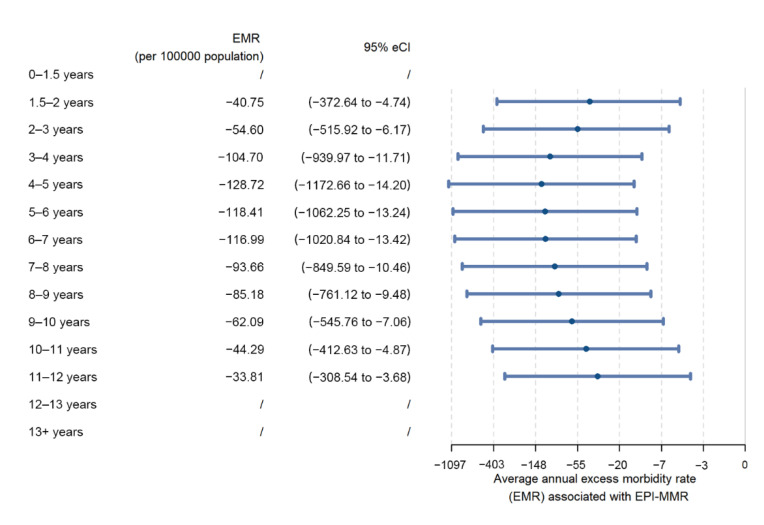
Average annual excess mumps morbidity rate associated with the EPI-MMR intervention in Guangzhou, China.

Figure 3 shows the number of mumps cases per 100,000 population per year for each age group that were estimated to have been prevented due to the EPI-MMR intervention. It seemed that the age-specific average annual EMR associated with the EPI was U-shaped, with the lowest average annual EMR occurring in the age group of 4–5 years, suggesting that an average of 128.72 (95% eCI: 14.20 to 1172.66) mumps cases per 100,000 population per year were averted due to the introduction of the EPI-MMR in this age group. In addition, it is evident from Figure 3 that the average annual EMR increased with age among those six years of age and older.

We obtained similar results from the sensitivity analyses. We found that whether the phase-in period was categorized as pre-intervention (CApre) or post-intervention (CApost), the incidence of mumps was significantly decreased, with a decrease of 38.6% (95% CI: 32.5% to 44.2%) and 38.3% (95% CI: 31.7% to 44.2%), respectively (Table S3). Whether a phase-in period was set or not, the introduction of the EPI-MMR reduced the incidence of mumps in all 1.5–12 years age groups for which the direct intervention was made (Table S4). Besides, two models were employed to examine the linear and potential non-linear changes in the intervention effects over time. The effect trends were graphically similar and the difference in the fitness of the two models was not statistically significant compared by the likelihood ratio test (*p* = 0.97), confirming that the change of effects over time was generally linear (Figure S5).

## 4. Discussion

Since September in 2008, Guangzhou has been including one dose of a combination of MMR for children aged 18–24 months in the EPI. Our findings indicated that the introduction of the EPI-MMR resulted in a reduction in the overall incidence of mumps in Guangzhou. In the 11 years following intervention, the auto-correlated mixed model revealed a 40% drop, excluding the effect of time-varying covariates in the mumps incidence rate. The number of mumps cases avoided due to the EPI-MMR was present in each age group. The findings shed some light on the modification of the mumps vaccination program, including a two-dose vaccination, and the expansion of the age range of the targeted population.

We acknowledge that single-site study has some limitations, while this study has the following strengths. First, mumps has been a notifiable disease and well-monitored in Guangzhou. All cases are mandatorily reported to local health authorities within 24 h via the National Notifiable Disease Surveillance System [25,26]. We can obtain high quality data from here. Second, Guangzhou is the largest city in China, except for Beijing and Shanghai. However, Beijing and Shanghai provided free two-dose mumps vaccination instead of one-dose vaccination in all other regions [14,27]; therefore, Guangzhou is representative to evaluate the effect of the EPI-MMR in China. Last, but not least, this study made some contributions to the methodology for evaluating public health intervention. We proposed a multi-age-group ITS design in which we treated each age group as panel data and set the starting time of intervention effect separately. This would provide a practical analytical strategy for evaluating the intervention effect of a public health policy, which is performed in a subpopulation (e.g., some specific age groups in this study), but which takes effect gradually on the whole population. We can estimate the effect for each age group simultaneously in a model by considering the possibility that the intervention group may be influenced by the non-intervention group. The time-series data from multiple age groups commonly present within-group autocorrelation and between-group correlations, especially for infectious diseases, and we found that this problem remains unsolved using ordinary models adjusting for various observed factors (Appendix A). To deal with the temporal autocorrelation within an age group, we defined the conditional covariance matrix for autocorrelation when estimating the parameters. Furthermore, we innovatively used the contact matrix of different age groups to capture the between-age-group correlations.

In practice, although the EPI-MMR was introduced in Guangzhou in September 2008, the number of mumps cases was not brought under control quickly and effectively. After a few years, it began to display a downward trend, similar to the situation observed in the whole of China, and in some other cities [28,29,30,31]. The reason for this is that from 2008–2011, only children aged 2–5 years were the target population of the EPI, while all children above five years are at high risk of mumps. Besides, the cluster 2 strains of genotype G strains first appeared in 2011, and were dominant in 2011 and 2012 [32]. Thus, the intervention effect was indiscernible in the first three years. However, the inclusion of children over five years of age in the model, who benefited from the EPI-MMR since 2011, has led to a significant decline in mumps incidence. This also suggests that we should set the starting time of the intervention separately for each age group, rather than analyzing the whole population directly, which would lead to unreasonable conclusions.

Some descriptive epidemiological approaches indicated the effectiveness of the EPI-MMR, although they failed to accurately quantify the effect of the intervention [30,31,33]. Some studies offered a third dose of MMR vaccine to some eligible students and calculated mumps incidence rates for specific periods, before and after the intervention [34,35]. However, it is difficult to draw conclusions about the effect of the intervention on the population as a whole. There is other evidence supporting the meaningful importance of mumps intervention in the fight against mumps. First, since the program was introduced, children’s MMR vaccination rates have gradually increased year after year, with coverage rates for two year olds exceeding 95% in several provinces and cities of China [27,36,37]. Second, a study in Shanghai showed a high seroprevalence of mumps antibodies has been achieved in urban regions; mumps antibody seroprevalence reached 90% (95% CI: 90.0% to 90.2%) when adjusted for age and gender after the introduction of the MMR vaccination [27].

Even though we estimated that the intervention was effective, mumps transmission did not completely disappear. We found that the effect became smaller (larger EMR) with increasing age for children above six years. This may be the relatively low incidence in the older age groups, even before the intervention. However, we must be aware of the vaccine’s diminishing effectiveness with time. Several studies found that following a single dose of MMR, immunity waned over time, and vaccinated children were still susceptible to mumps infection [38,39,40]. As a result, a two-dose mumps-containing vaccine schedule administered as part of normal services in China might aid in the prevention of mumps epidemics.

In the first year of the intervention, the effect was slight and even a non-significant increased risk was observed, especially when the phase-in period was categorized as post-intervention. This phenomenon indicates that it takes time for the policy to be implemented and for the vaccine to take effect. At the beginning, only children aged 1.5–2 years were vaccinated. All other children were at a high risk of mumps, and if they had mumps, they may infect the intervened populations because the vaccine is not 100% protective [41,42,43], which may mask the effect of the policy. From this point of view, the impact of the current high-risk population should be taken into account when developing policy implementation programs, and the age range of the intervened population should be appropriately expanded.

This study does have some limitations. To begin, we evaluated the effectiveness of the EPI-MMR intervention only in the city of Guangzhou, while we used contact data from Shanghai for analysis, due to data availability. More multi-site or nationwide studies would offer more reliable information to back up the promotion of the interventions in other places. Second, we can only calculate the direct effects due to the intervention in the model, which do not include spillover effects, thus underestimating the intervention effect to some extent. Last, due to incomplete records prior to 2005 and in order to exclude the impact of COVID-19 on the social distance, only a 11-year post-intervention period was involved in this study; this is not long enough to observe the long-term effect of the intervention, particularly in groups aged above 13 years.

## 5. Conclusions

We proposed a mixed-effects quasi-Poisson regression model in which the between-age-group correlations were artfully solved by using contact matrix of different age groups. This would be a novel analytical method for analyzing public health interventions across multiple groups. Our data strongly suggested that the introduction of the EPI-MMR effectively reduced the risks of mumps incidence in Guangzhou, China. The results would be useful in guiding the development, revision, and implementation of mumps preventive and control strategies.

## Figures and Tables

**Table 1:** Incidence rate and number of cases for yearly reported mumps in Guangzhou during 2005–2019.

Year	No. of Cases	Incidence Rate (per 100,000 Persons)
2005	8141	87.91
2006	6683	68.68
2007	3625	35.37
2008	4642	42.82
2009	4684	40.69
2010	5332	43.40
2011	10,500	82.50
2012	8318	65.01
2013	3867	30.02
2014	2443	18.79
2015	2602	19.58
2016	2329	16.91
2017	2651	18.58
2018	2521	17.15
2019	2344	15.52

## Data Availability

The data that support the findings of this study are available from the corresponding author upon reasonable request.

## Supplementary Materials

The following supporting information can be downloaded at: https://www.mdpi.com/article/10.3390/vaccines10101587/s1, Figure S1: the settings of phase-in period for each age groups; Figure S2: the results of the spectral analysis from January 2005 to December 2008 for each age group; Figure S3: the Moran’s I index for 14 age groups from January 2005 to December 2019 for the final model. Red points indicate there is significant between-groups correlation of residuals; Figure S4: the partial autocorrelation functions of residuals from the final model for 14 age groups; Figure S5: the linear (Line A) and potential non-linear (Line B) change of the effect of the EPI-MMR over time. The solid line indicates the point estimates of excess risks of mumps associated with the EPI-MMR and the shadow represents its 95% confidence intervals; Table S1: contact matrix of reported contacts for participants in Shanghai in 2019, consisting of the average number of contacts per day recorded by the survey participant; Table S2: the annual average incidence rate (per 100,000 population) for 14 age groups from 2005 to 2019; Table S3: excess risks of mumps incidence attributable to the EPI-MMR intervention in Guangzhou, China; Table S4: average annual excess mumps morbidity rate (EMR) associated with the EPI-MMR intervention in Guangzhou, China.

## Appendix A

In the preliminary analyses, we constructed an ordinary quasi-Poisson regression model as follows:

$$\begin{array}{ll} \log\left(u_{it}\right) & =a_{i}+offset\left(\log\left(pop_{it}\right)\right)+\beta_{1}t+\beta_{2}X_{it}+\beta_{3}t\times X_{it}\\ & +\overset{k}{\underset{\theta =1}{\sum }}\left[\beta_{1\theta }\sin\left(\frac{2\theta \pi mon_{m}}{T}\right)+\beta_{2\theta }\cos\left(\frac{2\theta \pi mon_{m}}{T}\right)\right]+ns\left(tem_{it},df=3\right)+ns\left(rh_{it},df=3\right)\\ & +ns\left(wind_{it},df=3\right)+ns\left(ssd_{it},df=3\right) \end{array}$$

where $u_{it}$ is the mean of the number of mumps cases in age group i in month $t\left(1,2,\ldots ,t_{s}-1,\ldots ,t_{e}+1,\ldots ,180\right)$, with a variance of $\varphi u_{it}$ conditional on $a_{i}$, the random intercept element that accounts for age-group heterogeneity. And $t_{s}$ is the start of phase-in period and $t_{e}$ is the end of phase-in period. And φ is the over-dispersion parameter. The logarithm of population, with a fixed regression coefficient of 1, was utilized as an offset. $X_{it}$ is a categorical variable with 0 and 1 expressing pre- and post-intervention period, respectively. The interaction term of $X_{it}$ and t expressed the time-varying effects of the EPI-MMR. To fit the seasonality of mumps incidence, the paired sine and cosine functions were applied; T is 12 and k was chosen by spectral analysis (k = 2) (Figure S1); $mon_{m}$ denotes the mth month of the year. $ns\left(tem_{it}\right)$, $ns\left(rh_{it}\right)$, $ns\left(wind_{it}\right)$, $ns\left(ssd_{it}\right)$ is natural cubic splines of monthly average temperature, average relative humidity, average wind speed and average duration of sunshine with three degrees of freedom (dfs).

Quasi-Poisson regression assumed independence of residuals within an age group and between age groups. However, this assumption was not validated since the partial autocorrelation function (PACF) of residuals revealed significant within-group autocorrelation at most age groups (shown in the Figure A1) and the Moran’s I index, in which the spatial matrix was replaced with the contact matrix, indicated significant between-group correlation (shown in the Figure A2).
