# Supplementary material for: A Multi-Age-Group Interrupted Time-Series Study for Evaluating the Effectiveness of National Expanded Program on Immunization on Mumps
Source: Vaccines (Basel). 2022 Sep 21;10(10):1587. doi: 10.3390/vaccines10101587 (PMC9610758; doi:10.3390/vaccines10101587)
Supplement: Supplementary file 1 [file vaccines-10-01587-s001.zip › vaccines-1900236-supplementary.pdf]

**Supplementary data**

Supplement to: SHI C, et al. A multi-age-group interrupted time-series study for evaluating the effectiveness of national Expanded Program on Immunization on mumps.

**Figure S1.** The settings of phase-in period for each age groups.

**Figure S2.** The results of the spectral analysis from Jan 2005 to Dec 2008 for each age group.

**Figure S3.** The Moran's I index for 14 age groups from Jan 2005 to Dec 2019 for the final model. Red points indicate there is significant between-groups correlation of residuals.

**Figure S4.** The partial autocorrelation functions of residuals from the final model for 14 age groups.

**Figure S5.** The linear (Line A) and potential non-linear (Line B) change of the effect of EPI-MMR over time. The solid line indicates the point estimates of excess risks of mumps associated with EPI-MMR and the shadow represents its 95% confidence intervals.

**Table S1.** Contact matrix of reported contacts for participants in Shanghai in 2019, consisting of the average number of contacts per day recorded by the survey participant.

**Table S2.** The annual average incidence rate (per 100,000 population) for 14 age groups from 2005 to 2019.

**Table S3.** Excess risks of mumps incidence attributable to EPI-MMR intervention in Guangzhou, China.

**Table S4.** Average annual excess mumps morbidity rate (EMR) associated with EPI-MMR intervention in Guangzhou, China.

**Figure S1.** The settings of phase-in period for each age groups.

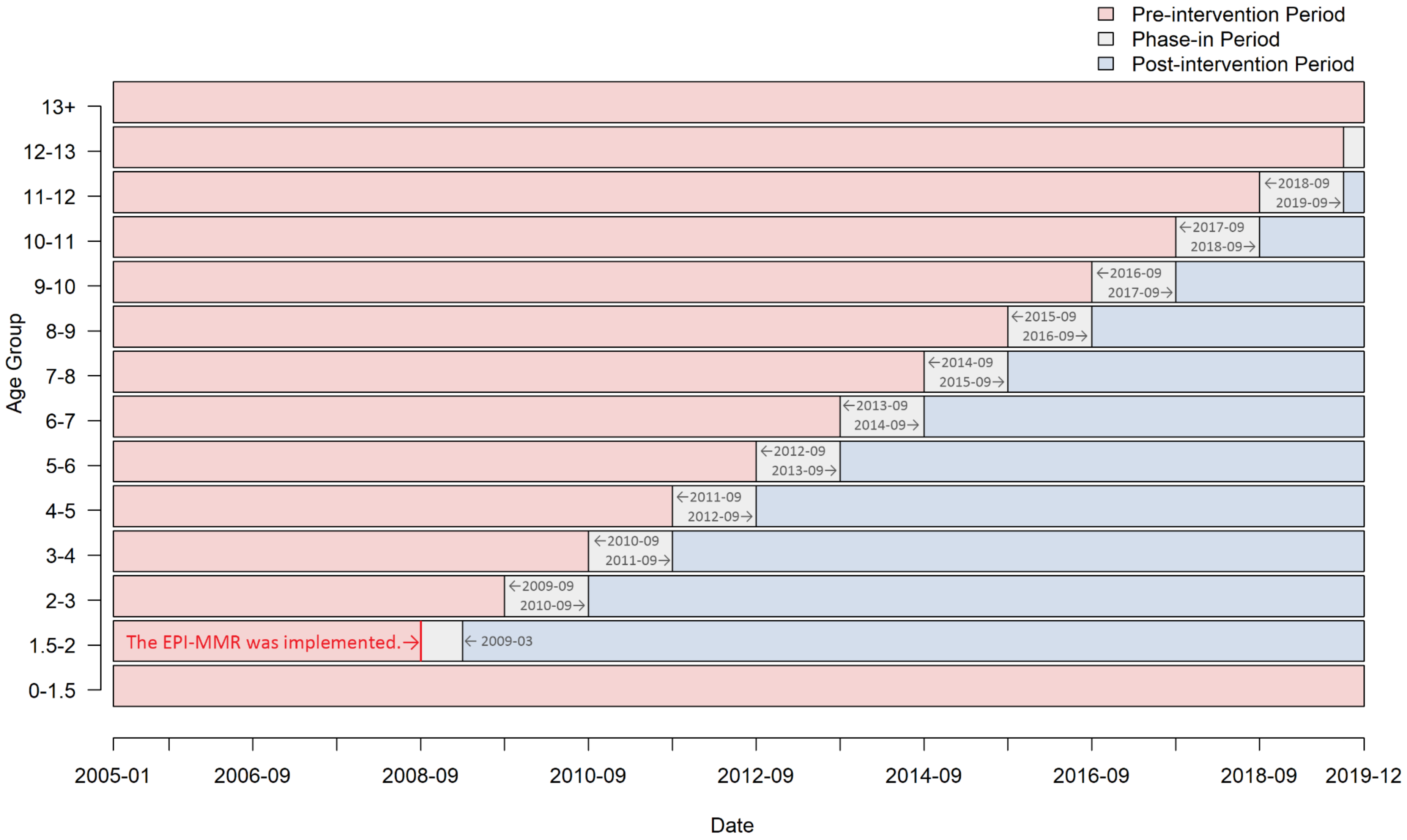

**Figure S2.** The results of the spectral analysis from Jan 2005 to Dec 2008 for each age group.

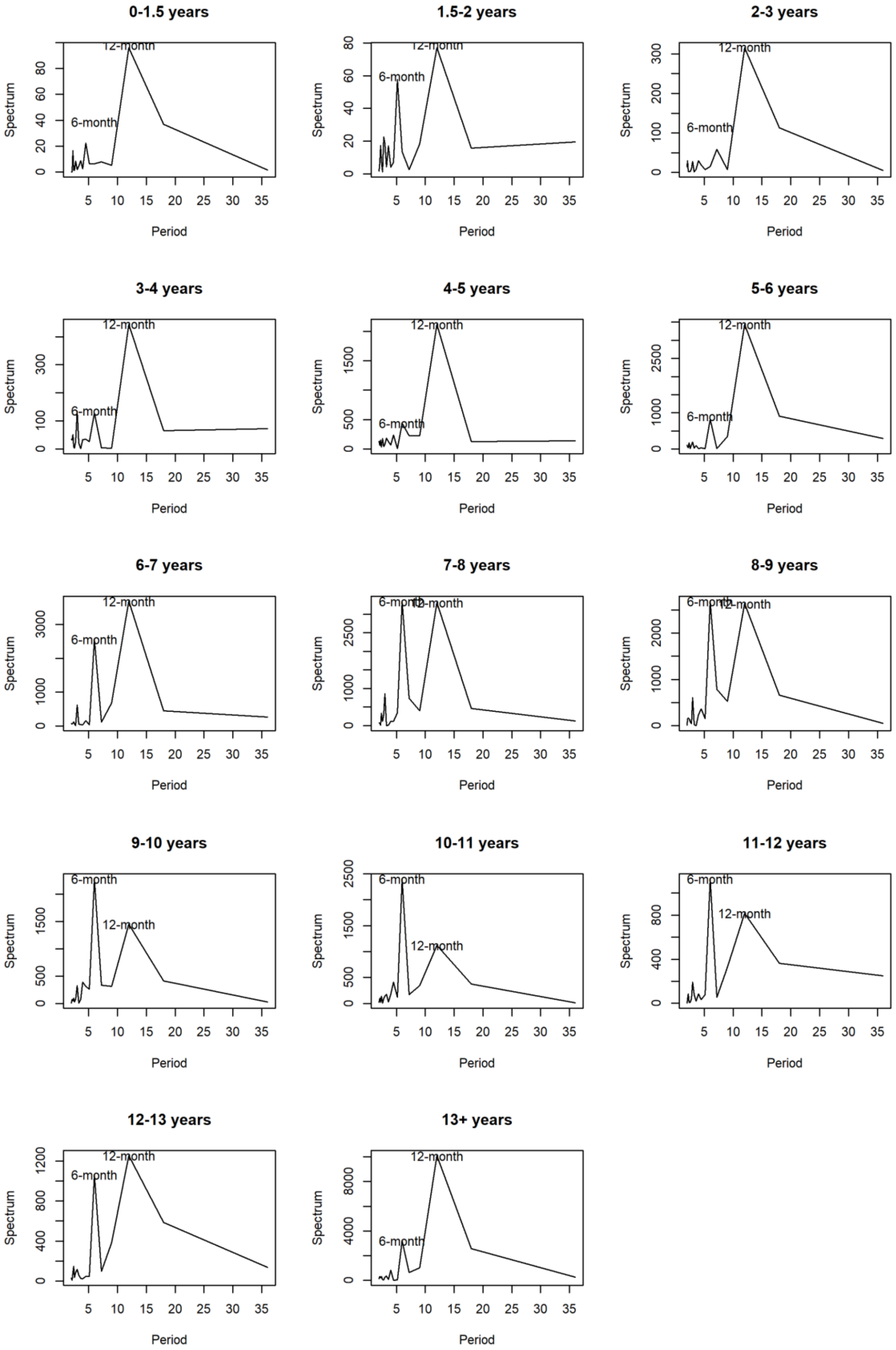

**Figure S3.** The Moran's I index for 14 age groups from Jan 2005 to Dec 2019 for the final model. Red points indicate there is significant between-groups correlation of residuals.

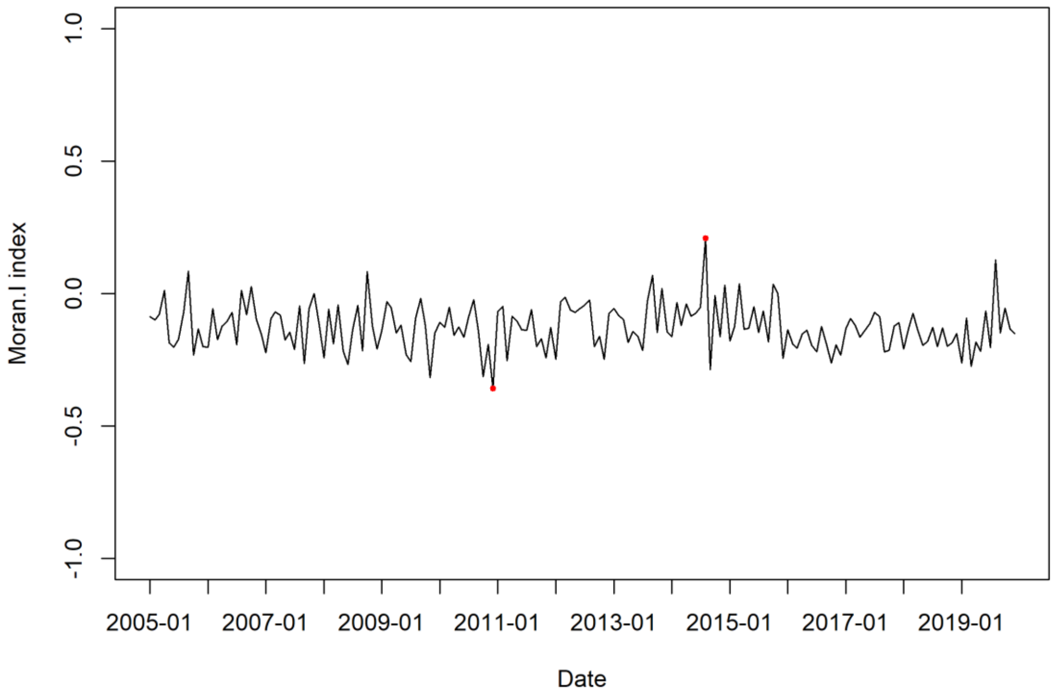

**Figure S4.** The partial autocorrelation functions of residuals from the final model for 14 age groups.

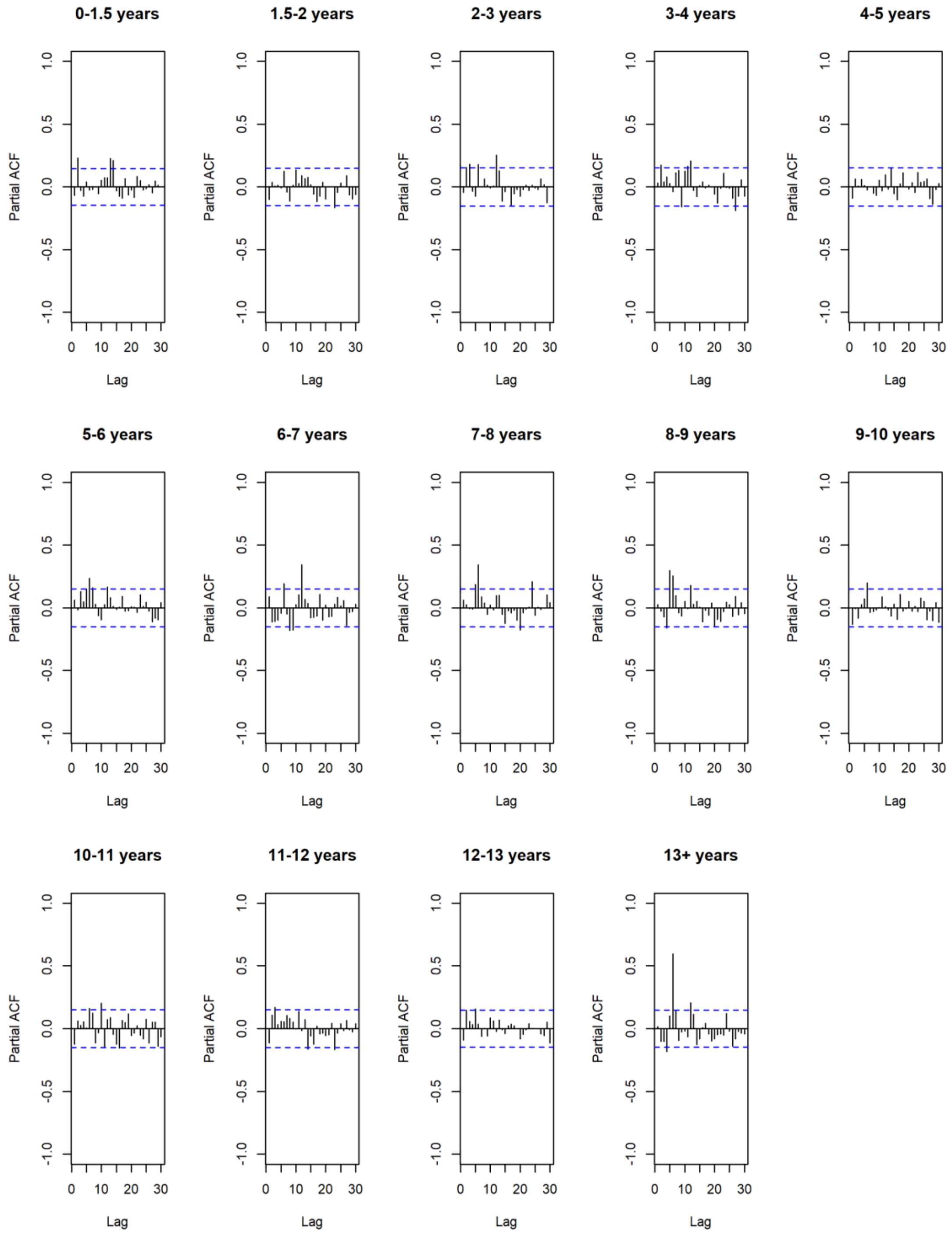

**Figure S5.** The linear (Line A) and potential non-linear (Line B) change of the effect of EPI-MMR over time. The solid line indicates the point estimates of excess risks of mumps associated with EPI-MMR and the shadow represents its 95% confidence intervals.

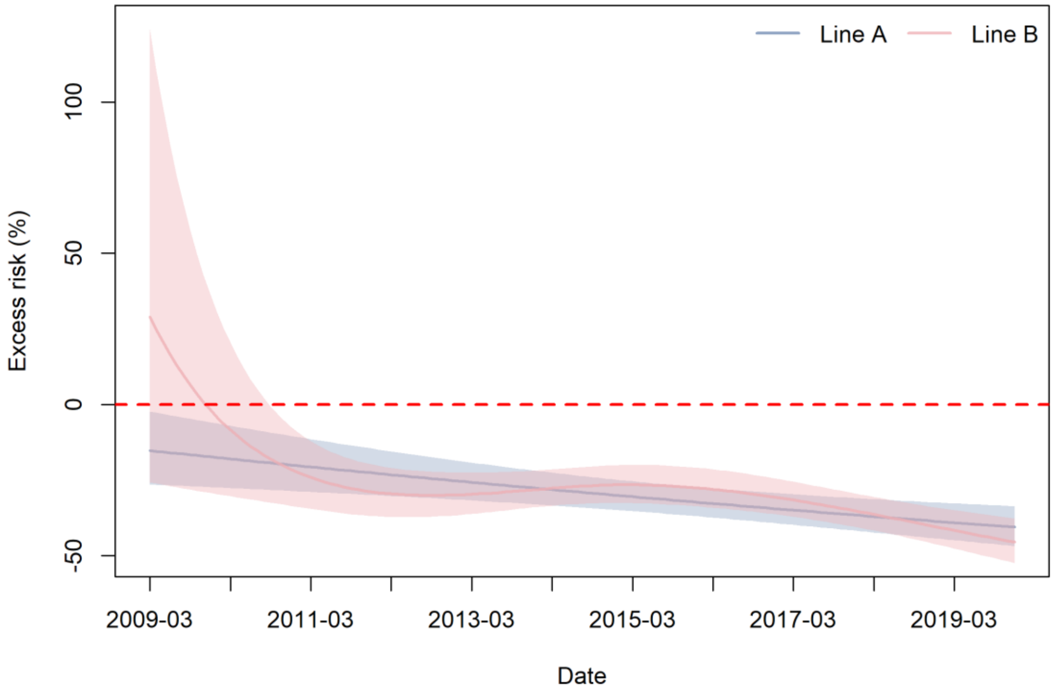

**Table S1.** Contact matrix of reported contacts for participants in Shanghai in 2019, consisting of the average number of contacts per day recorded by the survey participant.

| | | **Age of contact** | | | | |
|---|---|---|---|---|---|---|
| | | **[0,3)** | **[3,5)** | **[5,7)** | **[7,13)** | **13+** |
| **Age of participant** | **[0,3)** | 0.0811 | 0.0270 | 0.0270 | 0.0270 | 4.8378 |
| | **[3,5)** | 0.1071 | 0.3929 | 0.0714 | 0.0714 | 3.9286 |
| | **[5,7)** | 0.1053 | 0.2632 | 0.8421 | 0.2105 | 4.3684 |
| | **[7,13)** | 0.0169 | 0.0000 | 0.0169 | 2.1356 | 3.7797 |
| | **13+** | 0.0321 | 0.0508 | 0.0441 | 0.1591 | 6.6591 |

**Table S2.** The annual average incidence rate (per 100,000 population) for 14 age groups from 2005 to 2019.

| Year | 0-1.5 years | 1.5-2 years | 2-3 years | 3-4 years | 4-5 years | 5-6 years | 6-7 years | 7-8 years | 8-9 years | 9-10 years | 10-11 years | 11-12 years | 12-13 years | 13+ years |
|---|---|---|---|---|---|---|---|---|---|---|---|---|---|---|
| 2005 | 73.25 | 227.18 | 218.99 | 564.87 | 741.70 | 759.00 | 815.32 | 734.30 | 727.14 | 667.95 | 567.12 | 451.12 | 363.09 | 21.29 |
| 2006 | 61.35 | 253.40 | 253.66 | 523.48 | 665.67 | 703.73 | 706.53 | 536.72 | 546.37 | 487.61 | 415.19 | 377.41 | 330.18 | 15.65 |
| 2007 | 53.84 | 177.72 | 208.18 | 388.35 | 465.60 | 408.24 | 400.32 | 320.13 | 240.35 | 209.13 | 162.81 | 124.37 | 127.22 | 7.71 |
| 2008 | 51.01 | 146.87 | 196.86 | 459.19 | 554.90 | 441.95 | 472.47 | 503.48 | 376.98 | 296.85 | 244.66 | 148.12 | 117.41 | 10.60 |
| 2009 | 43.91 | 187.88 | 220.69 | 461.40 | 551.98 | 501.51 | 461.23 | 405.59 | 364.41 | 295.94 | 191.87 | 144.95 | 112.33 | 11.03 |
| 2010 | 43.89 | 169.35 | 223.01 | 548.59 | 733.45 | 604.86 | 548.82 | 453.84 | 411.44 | 308.72 | 241.43 | 196.26 | 143.81 | 10.10 |
| 2011 | 86.17 | 216.39 | 316.31 | 732.01 | 1233.79 | 1323.31 | 1093.39 | 972.49 | 826.38 | 613.88 | 523.05 | 391.03 | 303.73 | 20.97 |
| 2012 | 53.24 | 130.51 | 213.45 | 468.11 | 826.58 | 989.06 | 836.12 | 662.76 | 553.93 | 432.56 | 336.67 | 279.36 | 277.33 | 20.14 |
| 2013 | 40.54 | 106.58 | 134.81 | 221.45 | 330.49 | 425.40 | 355.35 | 287.04 | 251.19 | 193.55 | 139.67 | 114.22 | 83.43 | 9.20 |
| 2014 | 27.43 | 103.46 | 118.31 | 163.66 | 217.98 | 259.53 | 215.35 | 162.14 | 116.83 | 114.60 | 91.73 | 67.88 | 71.51 | 4.61 |
| 2015 | 32.81 | 78.22 | 119.59 | 188.44 | 252.43 | 236.35 | 193.97 | 174.10 | 156.34 | 128.34 | 99.39 | 74.12 | 51.86 | 4.03 |
| 2016 | 17.78 | 58.95 | 85.06 | 131.40 | 191.94 | 181.40 | 142.63 | 150.80 | 144.75 | 124.71 | 88.58 | 78.11 | 48.46 | 4.12 |
| 2017 | 18.71 | 53.78 | 73.26 | 123.70 | 169.75 | 217.39 | 170.55 | 119.30 | 148.68 | 131.12 | 108.90 | 83.77 | 61.25 | 5.19 |
| 2018 | 15.86 | 50.60 | 68.45 | 93.27 | 162.04 | 187.20 | 128.04 | 110.01 | 109.82 | 120.98 | 123.10 | 124.51 | 101.69 | 4.51 |
| 2019 | 12.86 | 37.28 | 72.99 | 86.72 | 134.38 | 137.68 | 132.37 | 109.89 | 82.10 | 76.20 | 94.94 | 102.85 | 97.34 | 4.49 |

**Table S3.** Excess risks of mumps incidence attributable to EPI-MMR intervention in Guangzhou, China.

| | ER [a] % (95% CI) | | |
|---|---|---|---|
| | phase-in period [b] | CApre [c] | CApost [c] |
| September 2009 | -16.61 (-27.02 to -4.71) | -13.64 (-24.66 to -1.02) | 3.34 (-7.86 to 15.90) |
| September 2010 | -19.32 (-28.25 to -9.27) | -16.54 (-25.99 to -5.88) | -1.85 (-11.30 to 8.61) |
| September 2011 | -21.94 (-29.55 to -13.50) | -19.33 (-27.36 to -10.42) | -6.77 (-14.71 to 1.89) |
| September 2012 | -24.47 (-30.94 to -17.40) | -22.04 (-28.80 to -14.63) | -11.45 (-18.13 to -4.23) |
| September 2013 | -26.93 (-32.49 to -20.91) | -24.65 (-30.36 to -18.47) | -15.90 (-21.63 to -9.75) |
| September 2014 | -29.30 (-34.24 to -23.99) | -27.17 (-32.09 to -21.89) | -20.12 (-25.24 to -14.65) |
| September 2015 | -31.60 (-36.26 to -26.60) | -29.61 (-34.08 to -24.84) | -24.13 (-28.99 to -18.94) |
| September 2016 | -33.82 (-38.53 to -28.75) | -31.97 (-36.34 to -27.30) | -27.94 (-32.83 to -22.68) |
| September 2017 | -35.97 (-41.00 to -30.52) | -34.25 (-38.84 to -29.31) | -31.55 (-36.70 to -25.99) |
| September 2018 | -38.05 (-43.56 to -32.01) | -36.45 (-41.48 to -30.99) | -34.99 (-40.50 to -28.97) |
| September 2019 | -40.06 (-46.13 to -33.32) | -38.58 (-44.16 to -32.45) | -38.25 (-44.18 to -31.70) |

[a] ER represents excess risk.

[b] phase-in period represents the ERs was calculated when a phase-in period was set.

[c] CApre and CApost represents the ERs was calculated when the phase-in period was categorized as pre-intervention and post-intervention, respectively.

**Table S4.** Average annual excess mumps morbidity rate (EMR) associated with EPI-MMR intervention in Guangzhou, China.

| Age group | EMR (95% eCI) per 100,000 population | | |
|---|---|---|---|
| | phase-in period [a] | CApre [b] | CApost [b] |
| 0-1.5 years | / | / | / |
| 1.5-2 years | -40.75 (-372.64 to -4.74) | -39.10 (-347.95 to -4.43) | -23.28 (-205.03 to -2.67) |
| 2-3 years | -54.60 (-515.92 to -6.17) | -53.02 (-497.60 to -6.01) | -33.86 (-317.15 to -3.89) |
| 3-4 years | -104.70 (-939.97 to -11.71) | -101.64 (-916.59 to -11.46) | -75.61 (-661.77 to -8.78) |
| 4-5 years | -128.72 (-1172.66 to -14.20) | -126.50 (-1140.89 to -13.81) | -108.71 (-953.31 to -12.29) |
| 5-6 years | -118.41 (-1062.25 to -13.24) | -116.15 (-1073.08 to -12.87) | -101.85 (-886.24 to -11.7) |
| 6-7 years | -116.99 (-1020.84 to -13.42) | -113.32 (-993.07 to -13.37) | -98.73 (-839.24 to -11.58) |
| 7-8 years | -93.66 (-849.59 to -10.46) | -91.20 (-813.40 to -9.97) | -80.13 (-697.45 to -9.24) |
| 8-9 years | -85.18 (-761.12 to -9.48) | -82.50 (-724.71 to -9.50) | -74.85 (-642.12 to -8.57) |
| 9-10 years | -62.09 (-545.76 to -7.06) | -60.58 (-511.66 to -7.02) | -58.49 (-497.83 to -6.83) |
| 10-11 years | -44.29 (-412.63 to -4.87) | -43.85 (-399.11 to -4.84) | -42.43 (-377.96 to -4.84) |
| 11-12 years | -33.81 (-308.54 to -3.68) | -34.20 (-310.65 to -3.82) | -32.83 (-288.91 to -3.66) |
| 12-13 years | / | / | -20.63 (-186.22 to -2.28) |
| 13+ years | / | / | / |

[a] phase-in period represents the EMRs was calculated when setting a phase-in period.

[b] CApre and CApost represents the EMRs was calculated when the phase-in period was categorized as pre-intervention and post-intervention, respectively.

/ represents the EMRs for these age groups cannot be calculated.
